# Editorial: Highlights in sport and exercise nutrition 2021/22

**DOI:** 10.3389/fspor.2023.1133147

**Published:** 2023-01-18

**Authors:** David C. Nieman, Stacy T. Sims, Laurel M. Wentz, Miguel Mariscal-Arcas

**Affiliations:** ^1^ North Carolina Research Campus, Human Performance Laboratory, Department of Biology, Appalachian State University, Boone, NC, United States; ^2^ WHISPA High Performance Sport New Zealand, Auckland, New Zealand; ^3^ Department of Nutrition and Healthcare Management, Appalachian State University, Boone, NC, United States; ^4^ Department of Nutrition and Food Science, School of Pharmacy, University of Granada, Granada, Spain

**Keywords:** sports nutrition, hydration, caffeine, dietary intake, chronic disease

Editorial on the Research Topic Highlights in sport and exercise nutrition 2021/22

This Research Topic features emerging research findings authored by leaders in the field of sport and exercise nutrition. The five papers in this Research Topic focused on hydration-related research, factors influencing dietary intake in rugby players, caffeine and athletic endeavor, and the influence of physical activity on chronic disease mortality rates.

The article “Estrogen to progesterone ratio and fluid regulatory responses to varying degrees and methods of dehydration” summarized data from a study investigating the relationship between fluid volume regulatory biomarkers and the estrogen to progesterone ratio (E:P) in response to dehydration in 10 women (Giersch et al.). One of these biomarkers was copeptin, which is a stable surrogate marker for arginine vasopressin (AVP) (1). AVP is a posterior pituitary hormone that has a direct antidiuretic action on the kidney. Copeptin and AVP are typically increased with fluid restriction and exercise. The data from this study showed that the relationships between copeptin concentration, estrogen, progesterone, and E:P following dehydrating exercise in the heat are weak and inconsistent. More information is needed on the role of E:P in body fluid regulation but these results do not show an impact of high estrogen with or without high progesterone on the copeptin response. The authors concluded that E:P hormonal fluctuations do not meaningfully regulate fluid responses during exercise in the heat and that women are not at a particular “disadvantage” during any phase of the menstrual cycle.

Another article, “Barriers, attitudes, and influences towards dietary intake amongst elite rugby union players”, focused on barriers that influenced optimal nutritional intake in a cross-sectional study of 30 high-level male rugby players (Sharples et al.). Childhood upbringing emerged as an important factor that influenced body composition and healthy eating habits. Body composition acted as both a barrier to and an enabler of optimal dietary intake. High-level rugby players relied on the team nutritionist, while those at lower performance levels were more influenced by the media and teammates. The authors concluded that there needs to be more nutrition education, with an emphasis on affordable food choices, meal planning, and skills for evaluating nutrition information obtained from the internet.

Caffeine use as an ergogenic aid is widespread. Most systematic reviews support the notion that, when used properly, caffeine from supplements and beverages improves endurance performance (2, 3). The paper “Caffeine supplementation strategies among endurance athletes” explored the findings from a cross-sectional study of 254 endurance athletes regarding the prevalence of caffeine use, dosing regimens, and perceived effects during training and racing (Kreutzer et al.). The survey indicated that 85% of respondents included caffeine in their diets but only 24% used caffeine supplements to improve performance. Low amounts of caffeine supplements were used and were taken closer to exercise bouts than typically recommended (i.e., 3–6 mg/kg body weight within approximately 60 min prior to exercise). The authors concluded that endurance athletes need better education regarding the amounts and timing of caffeine supplements.

The use of urine color charts to estimate hydration status has been supported in previous studies (4, 5). The validity of urine color charts has been challenged due to many confounding factors, such as the test environment, the urine container material, urine volume, subjective perception, age, and sex (6). The paper “Validation of urine color L*a*b* for assessing hydration amongst athletes” emphasized that urine color can be assessed in a more valid manner by using a spectrophotometer with color space models, such as L*a*b* (Feng et al.). The L*, a*, and b* values represent the change in brightness from black (0) to white (100), from green (−128) to red (+127), and from blue (−128) to yellow (+127), respectively. A total of 529 urine samples collected from 419 athletes were assayed for urine osmolality, urine-specific gravity, and urine color L*a*b* parameters using a benchtop spectrophotometer. Additional urine samples were collected from 55 athletes who submitted urine samples while drinking 100–200 ml of pure water every 10–20 min. The data revealed that urine color b* but not L* or a* was highly correlated with hydration status. As dehydration increased, urine color gradually turned yellow, with the b* value gradually increasing in a linear fashion along the blue-yellow axis. Another study focused on rapid dehydration and showed that both subjective and objective measures of urine color were more responsive than the use of urine osmolality and urine-specific gravity. There is growing support for the use of urine color b* to monitor both chronic and acute changes in hydration status in individuals engaging in intensive exercise bouts (6). This will require the support of a lab spectrophotometer and thus may be reserved for elite athletic settings.

Many epidemiological studies support the relationship between physical activity and a decreased risk for major chronic diseases in large populations followed for long periods of time (7). The paper “Dose-response association of leisure time physical activity with mortality in adults with major chronic diseases” took a closer look at the dose-response association between physical activity and all-cause and cause-specific mortality in adults with chronic diseases (Sun et al.). In this large prospective epidemiological study, adults with major chronic diseases had a reduced risk for all-cause, cardiovascular disease-, and cancer-specific mortality if they engaged in regular physical activity. Mortality rates dropped steeply up to 300 min/week of physical activity, with some further gains up to 600 min/week. The authors recommended that adults with chronic disease should be encouraged to engage in leisure-time physical activity, with workload volumes increasing according to capabilities. These findings are consistent with the “World Health Organization 2020 guidelines on physical activity and sedentary behavior” (7). These guidelines concluded that physical activity could confer health benefits for adults and older adults living with cancer, hypertension, cardiovascular disease, type-2 diabetes, and HIV. The WHO recommended that all adults and older adults with these chronic conditions should undertake regular physical activity.

## Author contributions

DN, SS, LW, and MM-A wrote this editorial and agreed to be accountable for the content of the work. All authors contributed to the article and approved the submitted version.
